# Aerial-hawking bats adjust their use of space to the lunar cycle

**DOI:** 10.1186/s40462-018-0131-7

**Published:** 2018-08-02

**Authors:** Manuel Roeleke, Tobias Teige, Uwe Hoffmeister, Friederike Klingler, Christian C. Voigt

**Affiliations:** 1 Leibniz Institute for Zoo and Wildlife Research, Alfred-Kowalke-Str. 17, 10315 Berlin, Germany; 2 Institut für Biologie, Freie Universität Berlin, Takustr. 6, 14195 Berlin, Germany; 3 Büro für faunistisch-ökologische Fachgutachten, Goldsternweg 34, 12524 Berlin, Germany; 4 Natura Büro für zoologische und botanische Fachgutachten, Hans-Sachs-Str. 48, 15732 Schulzendorf, Germany

**Keywords:** Flight altitude, Forest structure, Habitat use, LiDAR, Moonlight, *Nyctalus noctula*

## Abstract

**Background:**

Animals change their habitat use in response to spatio-temporal fluctuation of resources. Some resources may vary periodically according to the moonphase. Yet it is poorly documented how animals, particularly nocturnal mammals, adjust their use of space in response to the moonphase.

Here, we asked if an obligate nocturnal mammal, the aerial-hawking common noctule bat (*Nyctalus noctula*), adjusts its 3-dimensional flight behaviour and habitat use to the lunar period. Using miniaturized GPS loggers, we recorded 3-dimensional flight tracks of *N. noctula* and related these to a canopy height model derived from aerial laser scans to investigate whether bats adjust forest strata use to moonlight intensities.

**Results:**

Noctules frequently foraged above the canopy of coniferous forest at low moonlight intensities, but switched to using open grasslands and arable fields in nights with high moonlight intensities. During the few occasions when noctules used the forest during moonlit nights, they mostly restricted their use of space to flying below the canopy level. The median overall flight altitude of *N. noctula* equalled 13 ± 16 m but reached up to 71 m above ground (97.5% quantile).

**Conclusions:**

Our findings argue against general lunar phobic behaviour of aerial-hawking bats. We suggest that the preferred use of open fields around full moon may be a strategy of noctules to increase the success of hunting airborne insects at night. Specifically, the adjustment in use of space may allow bats to hunt for insects that emerge and disperse over open fields during bright moonlight.

**Electronic supplementary material:**

The online version of this article (10.1186/s40462-018-0131-7) contains supplementary material, which is available to authorized users.

## Background

Animals live in heterogeneous landscapes that offer resources for different requirements, such as breeding, shelter, or foraging [1]. Such functional heterogeneity within landscapes may occur in space and time alike [2]. At the spatial scale, animals will perceive the temporal heterogeneity of resource availability as a change in habitat suitability (cf [3]), which may result in distinct temporal patterns of use of space. Temporal changes in habitat suitability may be partially or completely unpredictable, e.g. when they are driven by local weather conditions [4], or distinct events like human hunting activities [5] or extreme weather conditions [6]. However, temporal changes in habitat suitability may also occur periodically. Periodic changes in habitat suitability and resulting changes in habitat use happen on very different timescales, ranging from hourly (e.g. tidal flooding [7]) to daily (e.g. day-night changes or periodic human disturbances [8]) and seasonal patterns (e.g. snow cover [9]). According to the optimal foraging theory [10], animals should react towards periodic and thus predictable temporal heterogeneity in habitat suitability with a concordant adjustment of their use of space.

The moon phase presents a highly predictable periodic change in the environment to which various animals respond. Many studies reported so-called lunar phobia in prey species, a term describing the negative response of animals towards bright moonlight by either decreasing overall activity [11, 12] or by adjusting habitat use and behaviour to prevent encountering visually oriented predators [11, 13–16]. Predators on the other hand may increase their activity during low or intermediate moonlight levels to enhance foraging success [17–19]. This may result in complex temporal and spatial patterns of predator-prey interactions [20]. Yet, some mammals are predator and prey at the same time, a fact that may result in a trade-off between increasing foraging activity when prey is easy to perceive, and decreasing activity at the same time in order to avoid becoming prey themselves during moonlit nights [21]. The optimal strategy thus depends on trading the energetic benefit from increased capture rate when hunting prey which is sensitive to the moonphase against the potential costs of increased predation risk in bright moonlight. One such strategy can be adjustment in use of space according to the anticipated resource distribution and likelihood of predation [21, 22].

Although bats are commonly perceived as lunar phobic animals [23], the picture within the order of bats is complex [24]. Thus far, lunar phobia has been described exclusively in some tropical bat species [25–27], yet with different reasoning for the underlying causes, such as predator avoidance or decreased prey detectability. On the other hand, studies on temperate zone bats could not show effects of moonlight on foraging activity [28–31]. However, although temperate zone bats might not decrease their overall flight activity, they may still adjust their vertical use of space, probably to increase foraging success [32]. This suggests that predation risk is only a minor driving force for temperate zone bats to alter their behaviour (cf [33]) and can be outweighed by the potentially higher foraging success during moonlit nights. Indeed, temperate zone bats face relatively small numbers of airborne predators during the night, and most aerial predators hunt only opportunistically upon bats when bats emerge from roosts at dusk [34–36]. Especially fast-flying bats that are adapted to forage in the open space [37] might be able to easily escape nocturnal birds of prey such as owls. This is probably also the reason why fast-flying species, like e.g. *Pipistrellus nathusii* or *Nyctalus noctula,* are the most light tolerant bats of the temperate zone (reviewed in [38]). Open-space foraging insectivorous bats of the temperate zone may thus be perceived as top predators. This will result in a high selection pressure to increase foraging efficiency, but a minor pressure to avoid predators. Bats might thus be highly flexible in their use of habitats and altitudes, enabling them to feed opportunistically on patches of prey aggregations, such as swarming insects. Indeed, many insects that hatch synchronously adjust their emergence to the lunar cycle [39, 40]. Some studies suggest a decrease of aquatic insects near full moon [41], whereas activity of terrestrial crop pests may increase with moonlight intensity [42]. These studies show that the timing of emergence is not consistent for all insect species, meaning that abundances of some insect prey species like specific moths may be low [40, 43] while the abundance of other insect prey species, e.g. Trichoptera or Diptera, may be high during the full moon [42, 44, 45]. Such species-specific responses towards the lunar cycle suggest temporal fluctuation of prey availability that is specific for the habitats that an affected prey species uses.

Here, we evaluate how the 3-dimensional use of space of common noctules (*Nyctalus noctula*) changes with moonlight intensity. *Nyctalus noctula* is a fast-flying species that forages in the open aerosphere [46], and is known for its flexibility in exploiting temporarily occurring and patchily distributed insect swarms (e.g. [47, 48]). Accordingly, if habitat specific insect abundances differ between moon phases, noctules should adjust their use of space to increase foraging efficiency. To test this hypothesis, we tracked common noctules with GPS loggers and related their habitat use and the use of forest strata derived from airborne laser scans (LiDAR) to the moon phase.

## Methods

### Study site and GPS tracking

In July 2015 and 2016, we equipped nine *Nyctalus noctula* (five post-lactating females and four males) with GPS loggers (Robin Cell Guide, Lucidlogix Technologies Ltd., Kfar Netter, Israel) to record 3-dimensional positions of flying bats. The study area in North-Eastern Germany consisted mostly of loose pine forest plantations interspersed by forest tracks (51%), but also included open fields (21%), several larger water bodies (14%), mixed or deciduous forest (8%), and small villages (5%) (Additional file 1). All tagged individuals roosted in artificial roost boxes in a pine stand, located about 50 km south of Berlin, Germany. During morning hours, we removed bats temporarily from their roosts and glued a GPS logger onto the dorsal fur of each bat using latex based surgical glue (Manfred Sauer, Lobbach, Germany). GPS loggers were placed into rubber balloons for protection against humidity. The whole unit weighed in total about 3.4 g, which corresponded to 10 to 13% of the bats’ body masses. Within a maximum of ten days after deployment of the GPS units, we relocated the tagged bats by using radio telemetry, recaptured them from their artificial roosting boxes or treeholes, and removed the GPS units. Similar to other studies [49, 50], we did not notice any adverse effects of the relatively large weight of GPS units on the bats. All procedures were approved by the animal welfare and ethics committee of the Landesamt für Umwelt, Gesundheit und Verbraucherschutz Brandenburg (permit: 2347–16-2015) and by the federal agency for nature conservation (permit: LUGV_N1–4743/103 + 5#283569/2015). All institutional and national guidelines for the care and use of animals were followed.

### Data acquisition and processing

We programmed the GPS loggers to record GPS locations every 15 s from sunset to sunrise until batteries expired. In total, we recorded about 7000 GPS locations from nine bats from which we retrieved GPS units. All bats started foraging trips around sunset, but only six bats performed additional trips within one night after times of inactivity. In these cases we divided the GPS locations of each bat into several continuous trips with regular GPS fix intervals, and deleted occasional GPS fixes when bats were not moving but remained in or close to their roosts. Further, we excluded flight times when - according to the three closest weather stations - more than 50% of the sky was covered by clouds, resulting in 22 flight trips (Table 1 and Additional file 2, 4929 GPS locations, between one and five trips or one and three nights per bat). Since altitude estimates of GPS loggers do not have the same accuracy as locations in the 2-dimensional plane, about 16% of locations yielded false negative flight altitudes at − 4.3 m (median) below ground. Most of these localisations were recorded when bats started their flight trips, flew within the forest, or when bats hunted above water bodies (see points with altitude zero in Additional file 1). Since the majority of these measures were thus recorded in situations when low flight altitudes are most plausible, we decided to off-set these points to zero and still include them in the analysis. We think that excluding these points from the analysis may have led to a severe overestimation of flight altitudes. However, one must be aware that the offsetting of false altitude measures leads to an underestimation of flight altitudes of localisations that are close to the ground or close to the canopy. Altitude measures of localisations further away from the ground or habitat structures on the other hand are measured at higher accuracy since satellite detection is not hampered at higher altitudes.

**Table 1:** Nights during which we tracked individual bats

Animal ID	Dates	No. of trips
A132503	02. July 2015	3
A132504	02. July 2015	1
A132518	02–03. July 2015	3
A132536	06. July 2015	2
A132726	11. July 2015	1
A132722	11. July 2015	1
A132542	16–18. July 2015	5
A132704	16–17. July 2015	5
A132670	28. July 2016	1

### Habitat use and movement behaviour

We assigned underlying land use types to the respective GPS locations using habitat maps derived from aerial infrared imagery [51] grouped into six categories: coniferous forest (i.e. mainly pine plantations), deciduous and mixed forest, open fields (incl. arable land, meadows, and grassland), urban areas, scrub or areas with successional growth, and water bodies or swamps. To evaluate the use of forest strata, we further assigned tree heights to the respective GPS locations when bats flew above the forest canopy. For this, we used aerial laser scan (LiDAR) data with a mean resolution of 2.9 points / m^2^ and an accuracy of < 20 cm, collected in 2009 by the federal office of the state of Brandenburg (https://www.geobasis-bb.de/geodaten/dgm-laserscanrohdaten.htm). Based on these raw data, we calculated a canopy height model (chm) for the forest areas, using the free version of the software LASTools (rapidlasso GmbH, Gilching, Germany) and following the tutorial by Isenburg [52]. A detailed description of the processing of the LiDAR data from raw 3-dimensional coordinates to the chm model is included in the supplement (Additional file 3). To assign the height of the uppermost canopy layer to the respective GPS points on a meaningful scale, we calculated the 95% quantile of the canopy height values within a radius of 20 m from the GPS location. For each GPS location, we specified the moonlight intensity as either low (0 to 20% of moon visible) or high (80 to 100% of moon visible). If the bats performed foraging trips before moonrise, we defined the according moonlight intensity as low. This resulted in flight tracks for four bats during high moonlight intensity in early July 2015 (one female and three males), and tracks from seven bats during low moonlight intensity in early and mid July 2015 and late July 2016 (4 females and 3 males) [Additional file 2].

We used the function fitHMM from the R package moveHMM [53] to assign two different movement behaviours (i.e. foraging with short step length and large turning angles, or commuting with larger step length and smaller turning angles) to single GPS fixes. Whenever the probability of correct classification was below 75%, we categorized the movement of a bat as undefined.

### Statistics

We used Mann-Whitney-U-tests to compare the flight altitudes of *N. noctula* between nights with high and low moonlight intensities above different habitats. To evaluate preferences for certain habitat types, we applied an use-versus-availability approach [54]. We defined available habitat for the respective tracks by five randomly rotated GPS tracks per recorded track (function NMs.randomShiftRotation, [55]) to keep the properties, such as the spatial autocorrelation structure, of the movement tracks [56]. The centre of rotation was set to the starting point of the respective track. We then fitted a binomial generalized mixed model with the interaction of habitat class and moonlight intensity as fixed factors to explain the identity of locations (i.e. real bat or randomly rotated track). We used the respective trips nested within the individual bat as a random factor to account for dependency of locations within single trips and between different trips of the same individual. We further included the sex of the tracked individuals as a random factor to account for potential biases in the dataset. We also modelled the probability of bats flying above or below the forest canopy, using a similar mixed model with only moonlight intensity as a fixed factor. Full models were compared to reduced models using Aikaike Information Criteria corrected for small sample sizes. We calculated 95% confidence intervals and plotted the fixed effects using the R package effects [57]. We assume statistical significant preference / avoidance when 95% confidence intervals did not overlap with a probability value of 0.5. For statistical tests, we assumed a significance level of 0.05. Unless stated otherwise, all measurements are given as median ± median absolute deviation (mad). Throughout the text, ranges are given as 2.5 to 97.5% quantiles. Data processing and statistics were done with the software R (Version 3.3.2). GPS data are stored at the movebank data repository (Study ID 297041945 at https:\\movebank.org).

## Results

### Moonlight and flight altitude

**Fig. 1:**
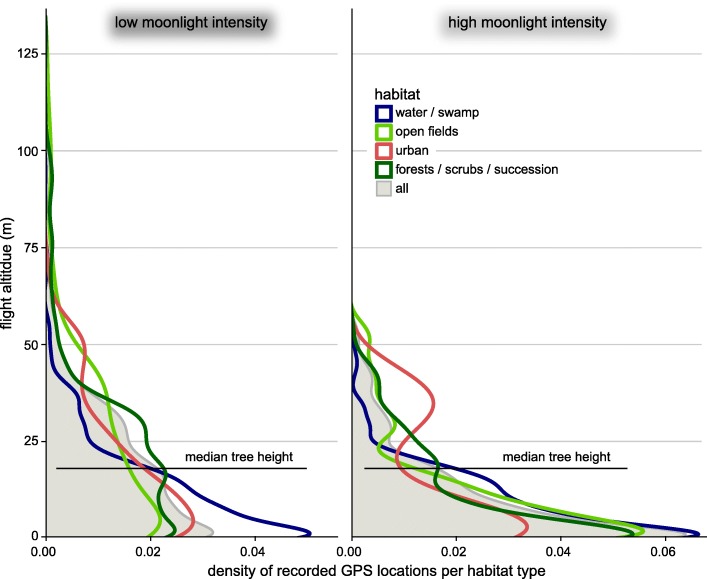
Probability of *N. noctula* flying above the canopy level when using forested areas, depending on the moonlight intensity. Dots depict effect estimates from the underlying model, bars depict the corresponding 95% confidence intervals

**Fig. 2:**
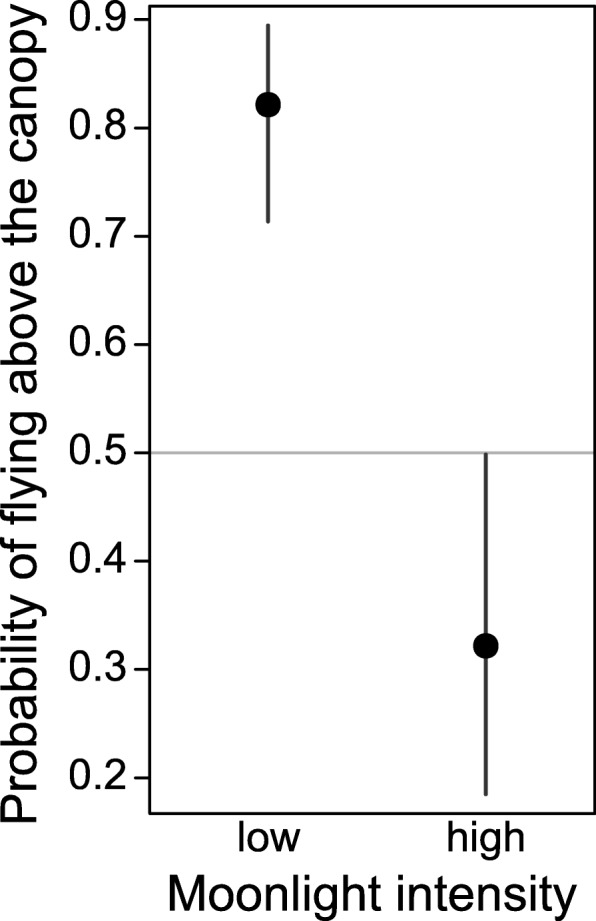
Relative distribution of flight altitudes of *N. noctula* for different habitat types and for all recordings, recorded at different moonlight intensities. The horizontal black line shows the median tree height, derived from all bat locations in forested areas

The median overall flight altitude of *N. noctula* equalled 13 ± 16 m (median ± median absolute deviation), but reached up to 71 m above the ground (97.5% quantile). This corresponded to a maximum altitude of 63 m above the canopy level (97.5% quantile) when noctules flew above forested areas In general, *N. noctula* flew at lower altitudes during high than during low moonlight intensities, except when flying above urban areas (Fig. 1, Table 1). *N. noctula* used forested areas less often during high than during low moonlight intensities (Table 2). When the bats used the forested areas during high moonlight intensities nonetheless, they flew mostly underneath the canopy level (Fig. 2).

**Table 2:** Flight altitude and relative time spend in different habitats during different moonlight intensities

	High moonlight intensity		Low moonlight intensity		
	Flight altitude (median ± mad)	Time spend in habitat	Flight altitude (median ± mad)	Time spend in habitat	Sig. diff. of flight altitudes
Water / swamps	6 ± 8 m	36%	8 ± 12 m	24%	yes, *p* < 0.001
Open fields	6 ± 10 m	29%	18 ± 23 m	9%	yes, *p* < 0.001
Forest / scrub / succession	6 ± 9 m	31%	18 ± 17 m	62%	yes, *p* < 0.001
Urban	7 ± 11 m	4%	13 ± 17 m	5%	no, *p* = 0.25
All	6 ± 9 m	100%	15 ± 17 m	100%	yes, *p* < 0.001

### Moonlight and habitat use

**Fig. 3:**
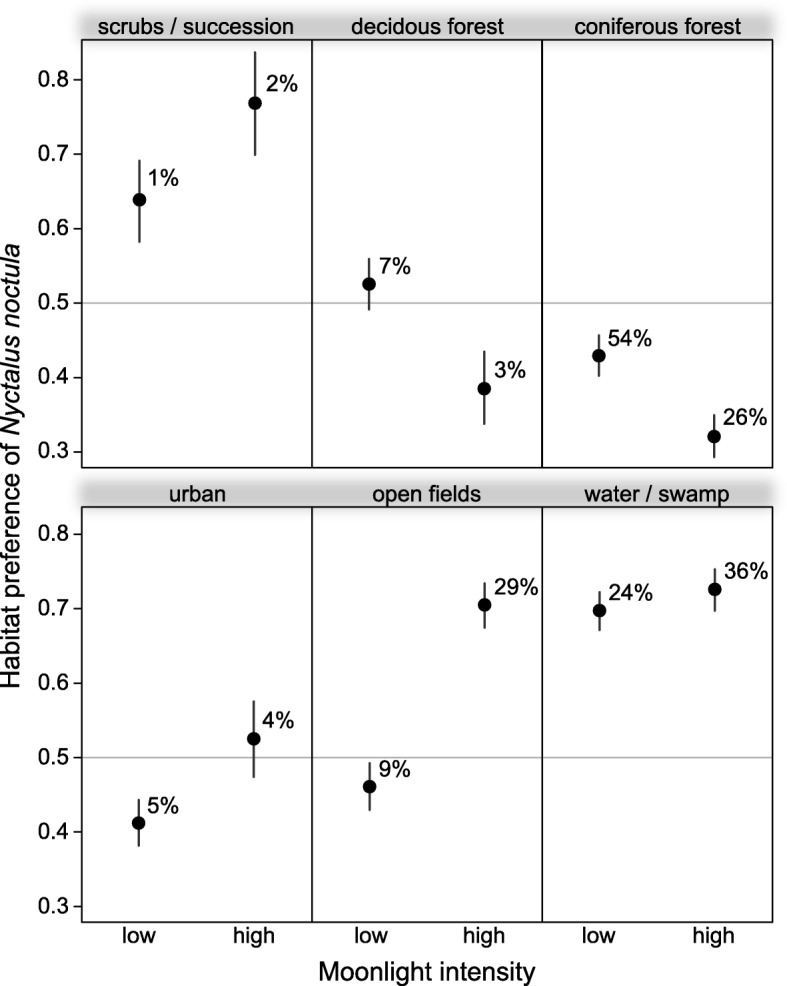
Preference of *N. noctula* for different habitat classes, depending on the moonlight intensity. Values above 0.5 indicate that *N. noctula* used this habitat more frequently than expected from availability derived from randomly rotated tracks. Values smaller than 0.5 indicate relative avoidance of the respective habitat type. Dots depict effect estimates from the underlying model, bars depict the corresponding 95% confidence intervals. Percentages depict the relative number of GPS locations within each habitat type for the respective moonlight intensity

**Fig. 4:**
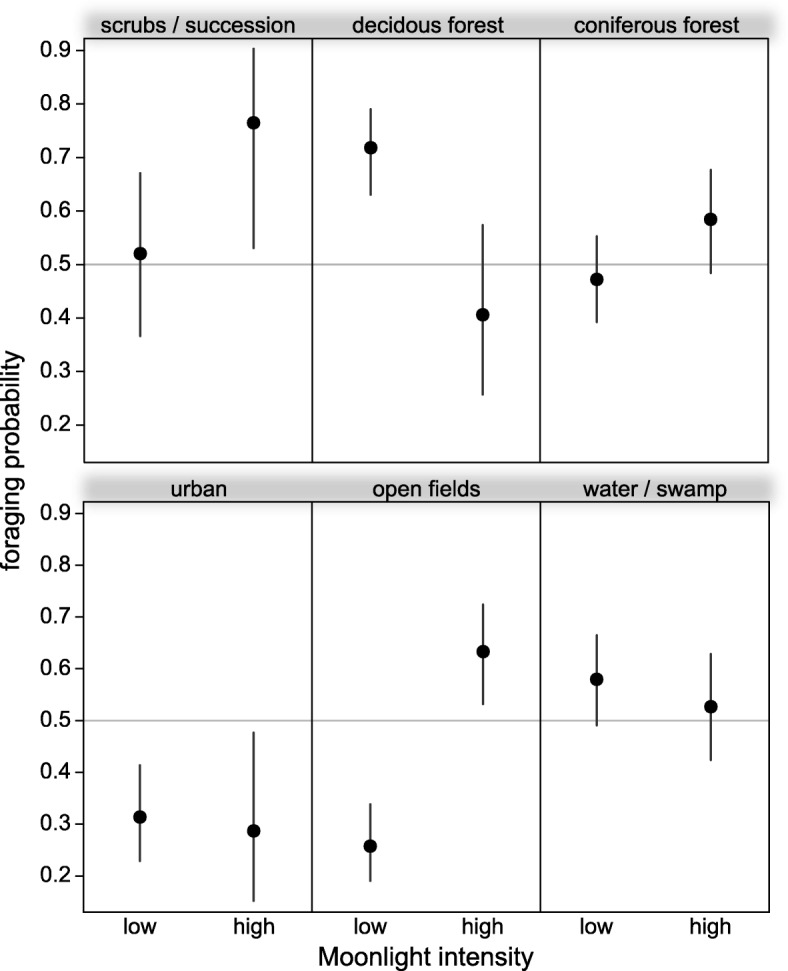
Probability that *N. noctula* showed movement behaviour associated with foraging, shown for the different habitat types and depending on the moonlight intensity. Values higher than 0.5 indicate that *N. noctula* used the respective habitat primarily for foraging during the given moonlight intensity. Dots depict effect estimates from the underlying model, bars depict the corresponding 95% confidence intervals

Irrespective of the moonlight intensity, *N. noctula* consistently preferred water bodies (Fig. 3). The recorded movement behaviour suggests that bats used the water bodies mainly for foraging (Fig. 4, in total 67% of the GPS locations over water were classified as foraging). At high moonlight intensities, noctules flew more often above open fields than at low moonlight intensities (Fig. 3). Their movement behaviour above open fields also suggests increased foraging activity under moonlit conditions (Fig. 4, 36% of GPS locations defined as foraging during low moonlight intensities, but 70% of GPS locations defined as foraging during high moonlight intensities).

*N. noctula* showed relative avoidance of coniferous forest at high moonlight intensities. Our model yielded also different significant effects of moonlight intensity on the use of urban areas, deciduous forest, and scrub or areas with successional growth (Fig. 3), as well as a significant decrease of movement behaviour associated with foraging activity in deciduous forest during high moonlight intensities (Fig. 4). However, these habitat types accounted only for a small fraction within the landscape, and since the GPS locations recorded in these habitat types only sum up to 12% of the total number of locations, we refrain from further interpretation of these results.

## Discussion

In early summer, we tracked nine common noctule bats (*Nyctalus noctula*) each for a period of several days in an area that was dominated by pine silviculture. Irrespective of the moonlight intensity, bats preferred water bodies for foraging, but also spent a considerable amount of time within or above the forest. However, during high moonlight intensities, bats used the forest less often but shifted their foraging activity towards open fields. When still using the forest during high moonlight intensities, bats tended to then fly under the shelter of the canopy level. *N. noctula* flew closer to the ground during high than during low moonlight intensities. It is intrinsic to the study setup that tracking during full moon and new moon cannot occur at the same time. However, we tracked all noctules (except one recorded in 2016) within two weeks in July 2015, a period of the year with constantly high insect abundance [58] and diversity [59], and without substantial changes in the annual life cycle of noctules [60, 61]. Further, there were no significant differences between ambient temperatures during the flights recorded at different moon phases (high moonlight intensity: 20.6 ± 3.6 °C, low moonlight intensity: 19.6 ± 4.0 °C, mean ± standard deviation) which might have influenced insect abundances. We are thus confident that the observed space use patterns are indeed related to moonlight intensities, and not confounded by the different days during which we tracked bats.

### Habitat use and the effect of moonlight

Waterbodies were the most preferred habitats for flight and foraging activity, followed by deciduous forests, and scrubland or successional areas. This is in accordance with a study by [62] which combined bat activity based on ultrasonic recordings with LiDAR data of forested area. In that study, long-range echolocating bats, such as *N. noctula*, were most active over rather heterogeneous areas, i.e. forest gaps and successional patches. However, since deciduous forests and successional areas were rare in our study area, the observed patterns for these habitats have to be interpreted with caution.

When noctules were foraging over waterbodies, they were least influenced by moonlight intensity. This is in concordance with former studies on habitat use of *N. noctula* [50, 63] and several dietary studies showing that *N. noctula* regularly feeds on aquatic insects [47, 64]. Insects hatching from the water surface are probably the most predictable food source for noctules in midsummer, irrespective of moonlight intensities. However, *N. noctula* is also known for its high dietary flexibility (reviewed by [48]), which explains the use of all available habitats within our study area.

We further found that the flight space above or within coniferous forests was overall used less often than expected from availability. The avoidance of coniferous forests was most pronounced during high moonlight intensities. When *N. noctula* nonetheless used the coniferous forest during high moonlight intensities, most GPS positions were recorded underneath the canopy level, and not above, as was the case when moonlight intensities were low. This is surprising since *N. noctula* is adapted to fly in uncluttered space at high forest strata [37, 65]. The flights underneath the canopy layer during both high and low moonlight intensities were probably mainly associated with roost searching and not foraging behaviour. A possible explanation for the lack of flights above the canopy during high moonlight intensities could be that foraging above the canopy at high moonlight intensities may not be beneficial enough for *N. noctula* to compensate for increased predation pressure from occasional bat-hunting birds of prey which are associated with the edge space between forests and open fields [66]. Alternatively, the lack of observations of noctules hunting above the forest canopy at high moonlight intensities might also simply be explained by the shift towards more profitable hunting areas, i.e. open fields.

However, one should be aware that altitude measures of bats that fly close to and especially underneath the canopy are suffering from reduced accuracy. Satellite signals blocked or reflected by vegetation or other structures and surfaces arrive with a delay and thus are more likely to result in falsely negative altitude estimates. Yet, the obvious breakpoint around the median canopy level height in the distribution of flight altitudes above forested areas makes us confident that the overall pattern of flight altitudes in relation to the canopy reflects the true behaviour of *N. noctula*.

Concurrent with decreased use of forest, activity of noctules above open fields and adjacent urban areas was highest during flight trips at high moonlight intensities. This finding is contradictory to the often proposed lunar phobic behaviour of bats (reviewed by [24]) associated with predator avoidance.

Indeed, some authors suggest that responses of bats towards the moon phase may most likely be driven by prey availability (e.g. [25]). Hecker and Brigham [32] found that under moonlit conditions, some bat species (mainly belonging to the genus *Myotis)* shifted their hunting grounds from lower strata of the forest to the canopy level. They conclude that prey availability rather than predator avoidance may be the driving factor. This is supported by Speakman et al. [67], who found that bats continued their night activity patterns in the Nordic summer, despite bright conditions during the whole night and despite higher prey availability at daytime. They conclude that night activity of bats in temperate zones may have evolved to avoid competition with birds, but not to reduce predation pressure. This is supported by Voigt and Lewanzik [68] who suggest that during daytime, flight costs for bats are considerably higher than for birds, and another study by Speakman and Webb [69] showing that *Nyctalus azoreum* primarily forages at night time, although avian predators are not present in its habitat. Indeed, dietary studies on night active birds of prey such as owls indicate that bats comprise only a minor fraction of their prey [34–36], but this might vary geographically [70] and seasonally [71]. Despite the low fraction of bats in the diet of predators, Speakman [72] estimated that birds of prey may still account for 10% of the mortality of bats in Britain. Based on that estimate, one would assume that also temperate bats are under strong selection pressure to avoid predators. Our results on the effect of moonlight on the activity of *N. noctula* appear inconsistent with lunar phobia being caused by predator avoidance. The tracked bats exhibited a behaviour which is better explained by the term lunar philia, since they shifted their use of space towards open fields under moonlit conditions. In this context, lunar philia has to be understood as an active habitat choice towards landscapes where bats are exposed to moonlight under bright conditions, without any a priori implications of the underlying reasons. Our findings suggest that predator avoidance is not causative for the observed pattern, probably because noctules are not as vulnerable to predation as slow flying bat species. On the other hand, when using the forest under moonlit conditions, noctules switched from flying above to flying underneath the canopy. Since noctules are not adapted to forage within dense forest, the reason might have been to avoid being spotted against the moonlit sky by predators ambushing from exposed tree branches. This may partially also explain the lower flight altitudes of *N. noctula* when foraging above open fields under moonlit conditions. Being an opportunistic forager [48], *N. noctula* seems to be able to shift its habitat use in response to moonlight in such a manner that it optimizes the exploitation of cyclic appearing insects while minimizing predation risk by adjusting their flight altitude and avoiding habitats with temporarily high predation risk.

Such a temporal plasticity in habitat use is supported by the finding the *N. noctula* not only spend more time above open fields, but also increased the relative amount of foraging behaviour above open fields during high moonlight intensities. We thus speculate that prey availability above open fields increases under moonlit conditions, turning open fields regularly into valuable foraging habitats for open-space foraging bats, and compensating for potentially increased predation pressure, at least for fast-flying bats like *N. noctula*. Unfortunately, literature on the effect of moonlight on insect abundances is contradictory. Reduced insect activity under moonlit conditions was reported in the early literature and yet later contradicted by some authors [73, 74], but other studies support the idea of moonlight avoidance by insects [41]. Some authors on the other hand suggest that emergence of insects is synchronized by the moon phase, with the timing of emergence being species-specific but most often associated with near full moon [39, 40]. However, most studies on insect abundance so far used light-traps, a method that likely is biased during high moonlight intensities. Yela and Holyoak [75] showed that light-traps were less efficient for catching noctuid moths in forests during high moonlight intensities, while catches from bait traps were not influenced by moonlight. Using light-traps, [44] caught more Lepidoptera under dark conditions and more Hemiptera under bright conditions. When investigating the diet of *Myotis sodalis*, they found a higher portion of Diptera and aquatic insects, and a lower portion of Lepidoptera with increasing moonlight. It remains unclear whether this shift in the diet could be attributed to shifting insect availability or to a shift in habitat selection by bats. Contrary to that, a study by [43] showed that especially open-habitat associated moths as well as dipteran species may be most active during moonlit nights. Bidlingmeyer [45] found that abundance of different mosquito species increased with moonlight when sampling with funnel traps on roads near a beach. This indicates that mosquitoes may synchronize hatching close to full moon and then distribute over the landscape. Overall, evidence is accumulating that many insect taxa synchronize hatching to the moonphase, yet without a consistent pattern for the exact time. This species-specific timing must thus result in different insect densities at the respective habitats of the insects, leading to temporal heterogeneity in habitat suitability for insectivorous predators. Especially light tolerant species such as *N. noctula* and other open space foraging bats [38, 76] may be able to exploit such insect rich open habitats despite intense moonlight. Further, a study by Eklöf et al. [77] showed that open space foraging bats use vision when hunting for moths in cluttered habitats, a fact they may have enhanced the foraging success of *N. noctula* when hunting at the edges of open fields during high moonlight intensities.

Yet, we must acknowledge that due to ethical and technical constraints, our study period was limited to the post breeding season. It might thus be that the observed responses towards moonlight levels may change throughout the season, e.g. when female bats are raising young and may thus be more risk sensitive towards potential predation.

## Conclusions

This study confirms that predators such as insectivorous bats can be highly flexible in their use of space, probably in order to increase foraging efficiency by exploiting temporarily occurring prey accumulations. The shift of *N. noctula* from forested to open fields during high moonlight intensities argues against the notion that bats generally exhibit lunar phobia as a predator avoidance strategy and thus hide during moonlit nights. We speculate that some bat species actively chose open fields under moonlit conditions to exploit insects that are lured out of the vegetation when moonlight intensities are high. Yet, predator avoidance behaviour may explain decreases in bat activity in temporarily risky spaces, such as the space above the canopy of forested areas. Irrespective of the underlying reasons, the observed change in use of space highlights that habitat suitability is not static for bats and other nocturnal animals but may shift periodically in response to the lunar phase.

## Additional files

Additional file 1: Habitat types within the study area. The location of the artificial roosts is indicated by the white star. (PNG 3728 kb)

Additional file 2: Flight altitude for all recorded tracks. Each dot represents one GPS location, whereas colour depicts whether the observed movement behaviour was associated with foraging. Green ribbons depict the underlying canopy height in forested areas. Background colours depict the different habitat types; blue = water /swamp, red = urban, light-green = open fields, dark-green = forests and scrub / successional growth. The colour of the horizontal bar on top of each trip depicts the moonlight intensity; black = low, yellow = high. (PDF 2338 kb)

Additional file 3: Workflow to create the canopy height model by using the lastool software. (TXT 2 kb)

## Abbreviations

chm: canopy height model
GPS: Global positioning system
LiDAR: Light detection and ranging
