# Supplementary figures and images for: Aerial-hawking bats adjust their use of space to the lunar cycle
Source: Mov Ecol. 2018 Aug 2;6:11. doi: 10.1186/s40462-018-0131-7 (PMC6090956; doi:10.1186/s40462-018-0131-7)

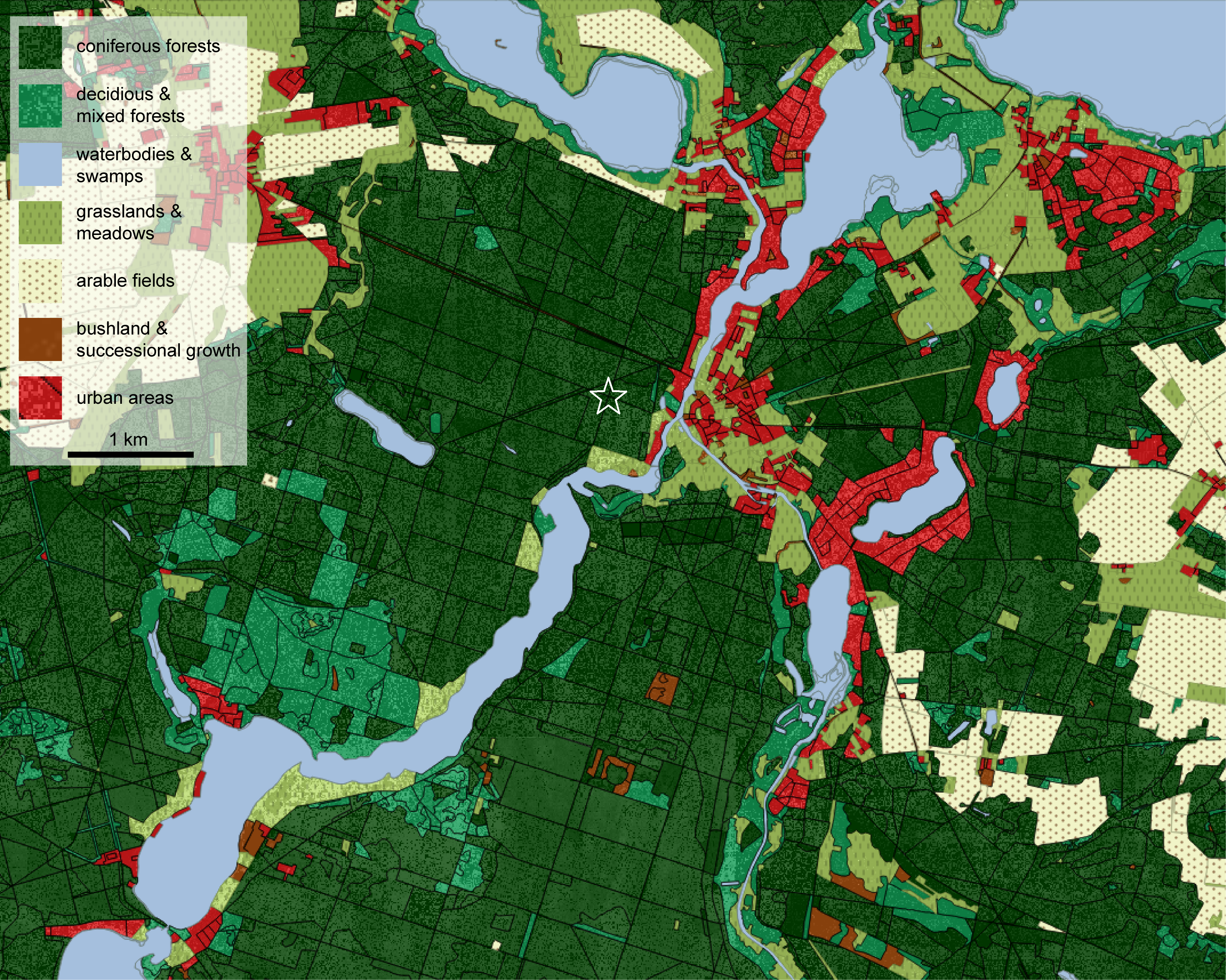

Supplement: Supplementary file 1 — Habitat types within the study area. The location of the artificial roosts is indicated by the white star. (PNG 3728 kb) [file 40462_2018_131_MOESM1_ESM.png]
