# Supplementary material for: Aerial-hawking bats adjust their use of space to the lunar cycle
Source: Mov Ecol. 2018 Aug 2;6:11. doi: 10.1186/s40462-018-0131-7 (PMC6090956; doi:10.1186/s40462-018-0131-7)
Supplement: Supplementary file 2 — Flight altitude for all recorded tracks. Each dot represents one GPS location, whereas colour depicts whether the observed movement behaviour was associated with foraging. Green ribbons depict the underlying canopy height in forested areas. Background colours depict the different habitat types; blue = water /swamp, red = urban, light-green = open fields, dark-green = forests and scrub / successional growth. The colour of the horizontal bar on top of each trip depicts the moonlight intensity; black = low, yellow = high. (PDF 2338 kb) [file 40462_2018_131_MOESM2_ESM.pdf]

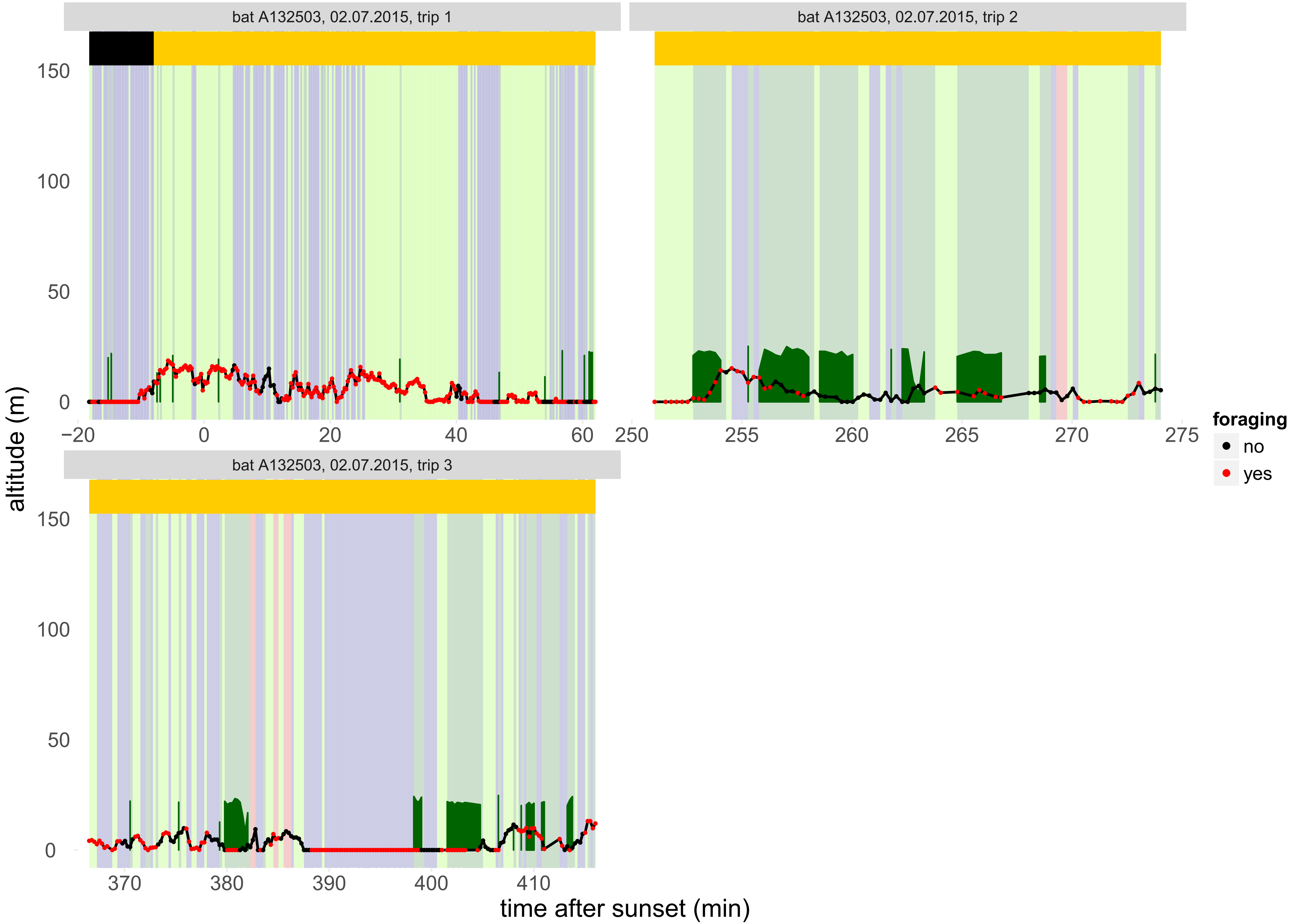

bat A132503, 02.07.2015, trip 1
bat A132503, 02.07.2015, trip 2
bat A132503, 02.07.2015, trip 3
altitude (m)
150
100
50
0
−20
0
20
40
60
250
255
260
265
270
275
370
380
390
400
410
time after sunset (min)
foraging
no
yes

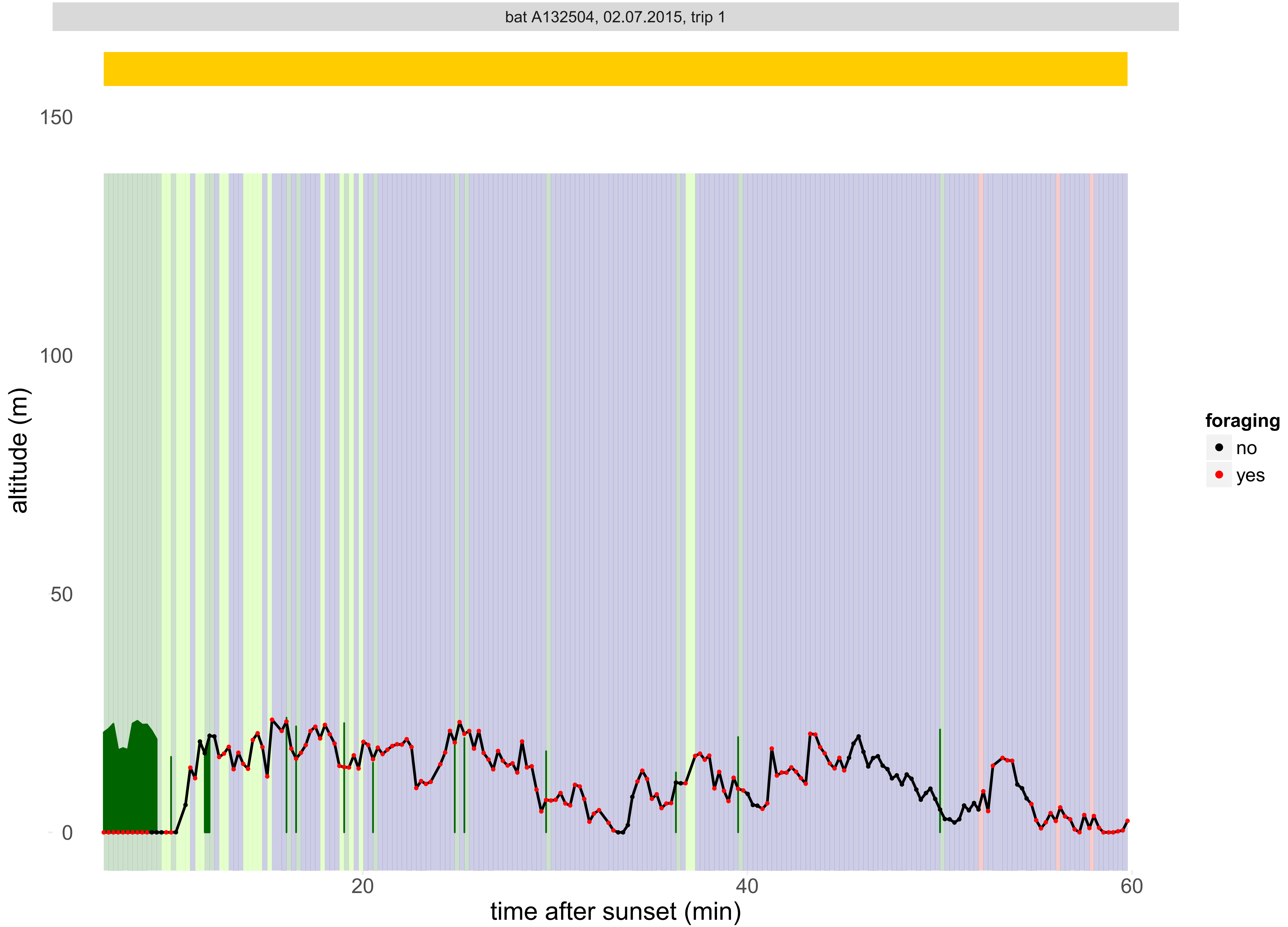
bat A132504, 02.07.2015, trip 1
150
100
50
0
altitude (m)
20
40
60
time after sunset (min)
foraging
no
yes

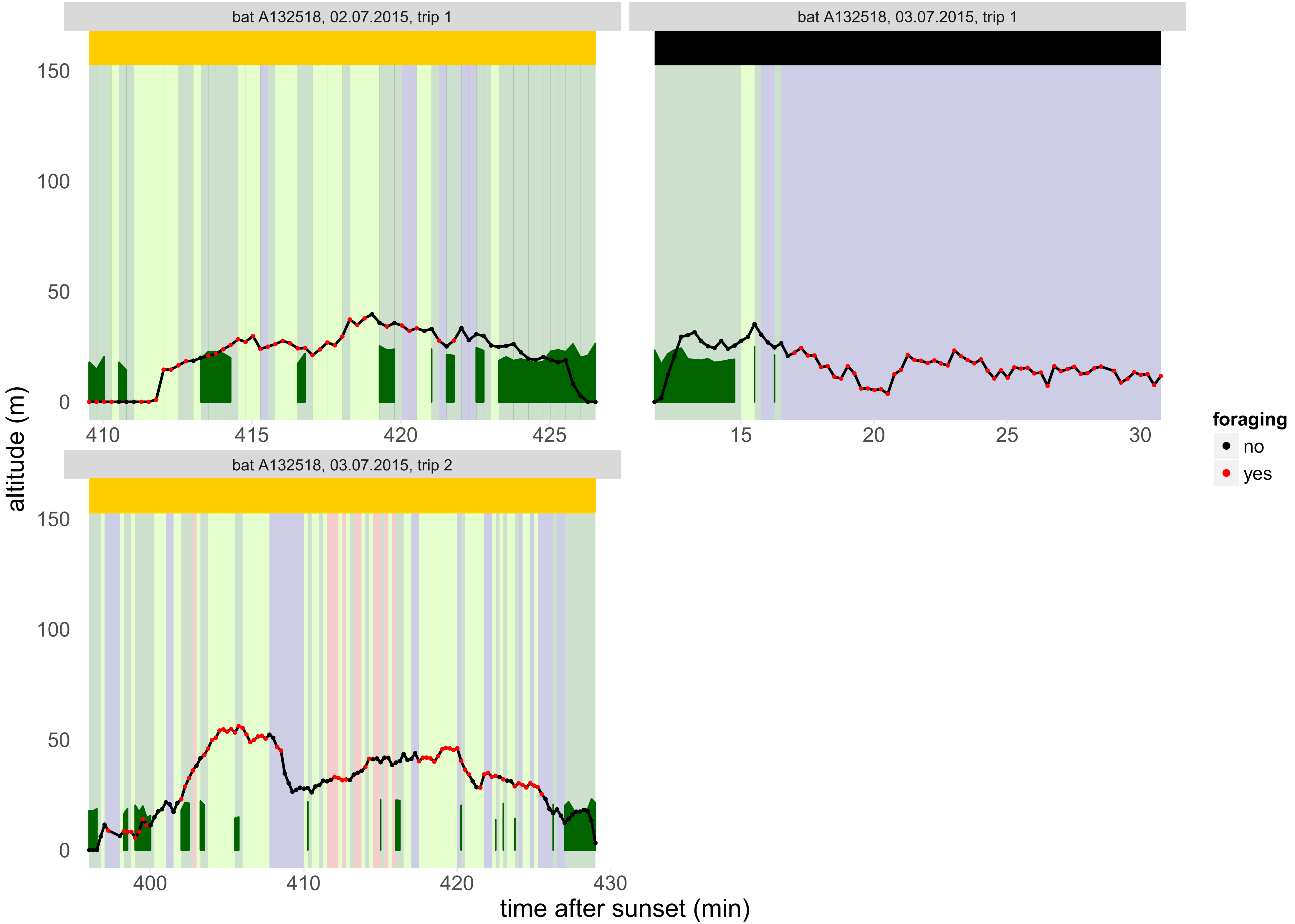

bat A132518, 02.07.2015, trip 1
bat A132518, 03.07.2015, trip 1
bat A132518, 03.07.2015, trip 2
altitude (m)
time after sunset (min)
foraging
no
yes
0
50
100
150
410
415
420
425
15
20
25
30
400
410
420
430

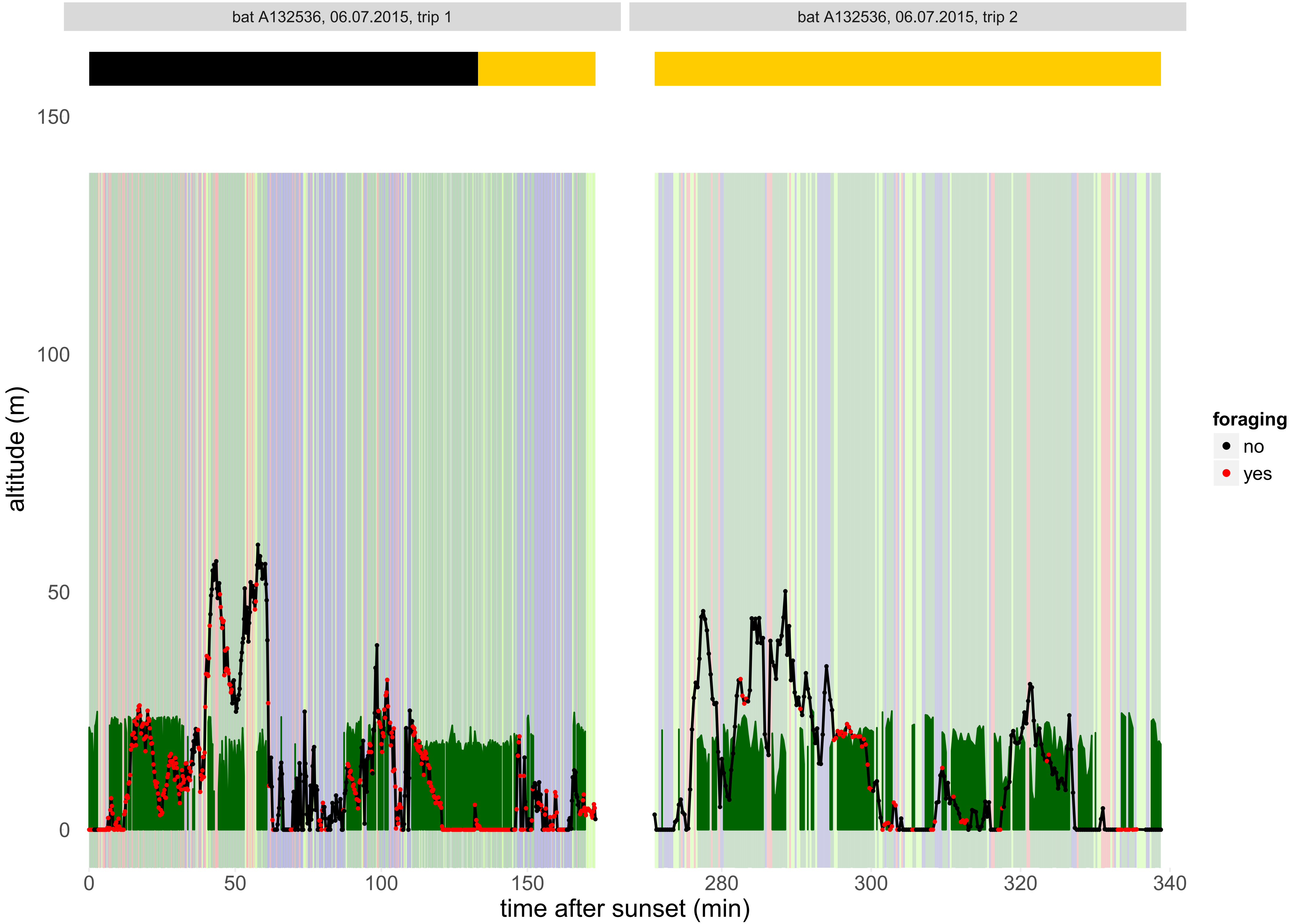
bat A132536, 06.07.2015, trip 1
bat A132536, 06.07.2015, trip 2
altitude (m)
150
100
50
0
0
50
100
150
280
300
320
340
time after sunset (min)
foraging
no
yes

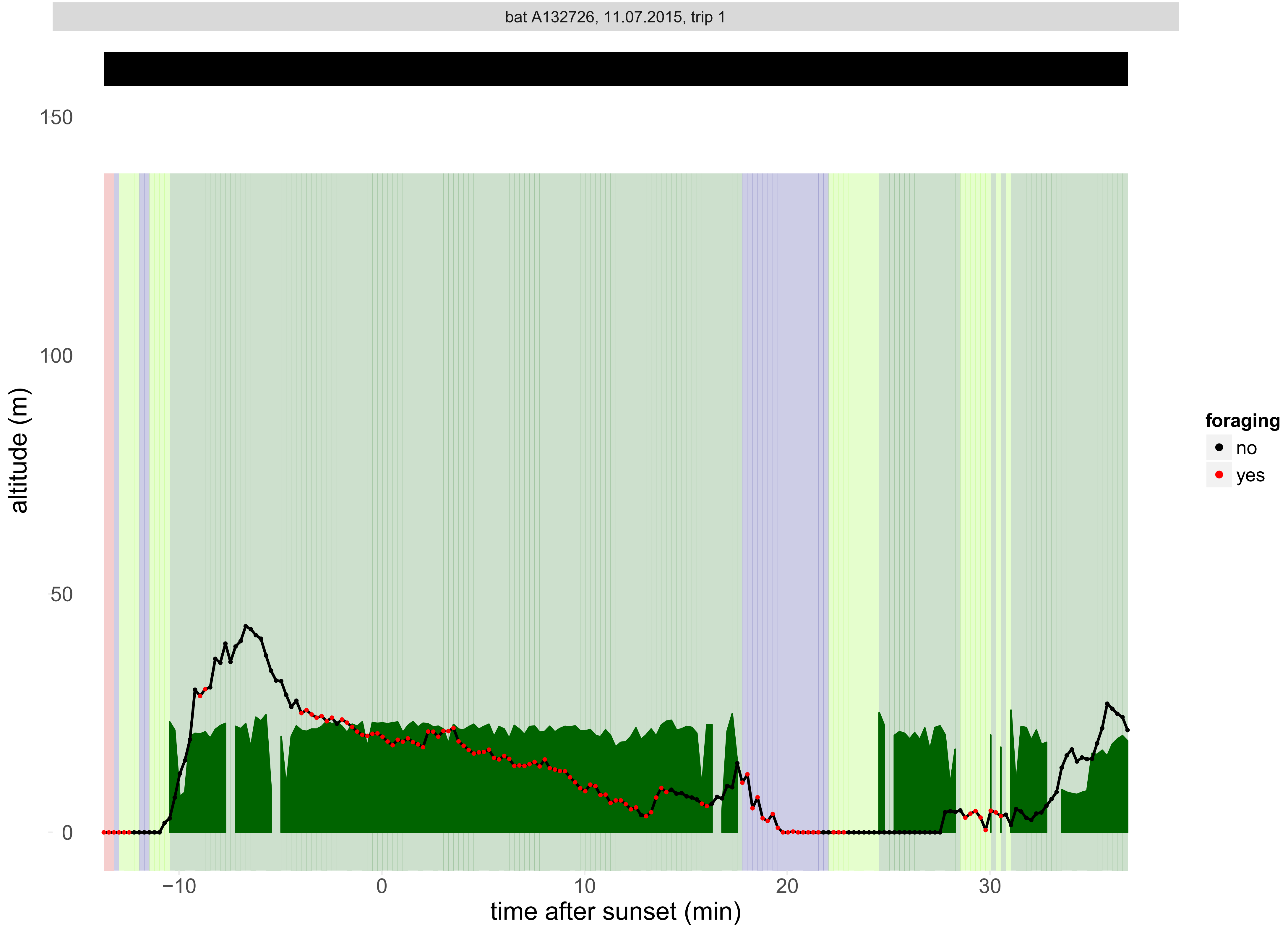
bat A132726, 11.07.2015, trip 1
150
100
50
0
altitude (m)
−10
0
10
20
30
time after sunset (min)
foraging
no
yes

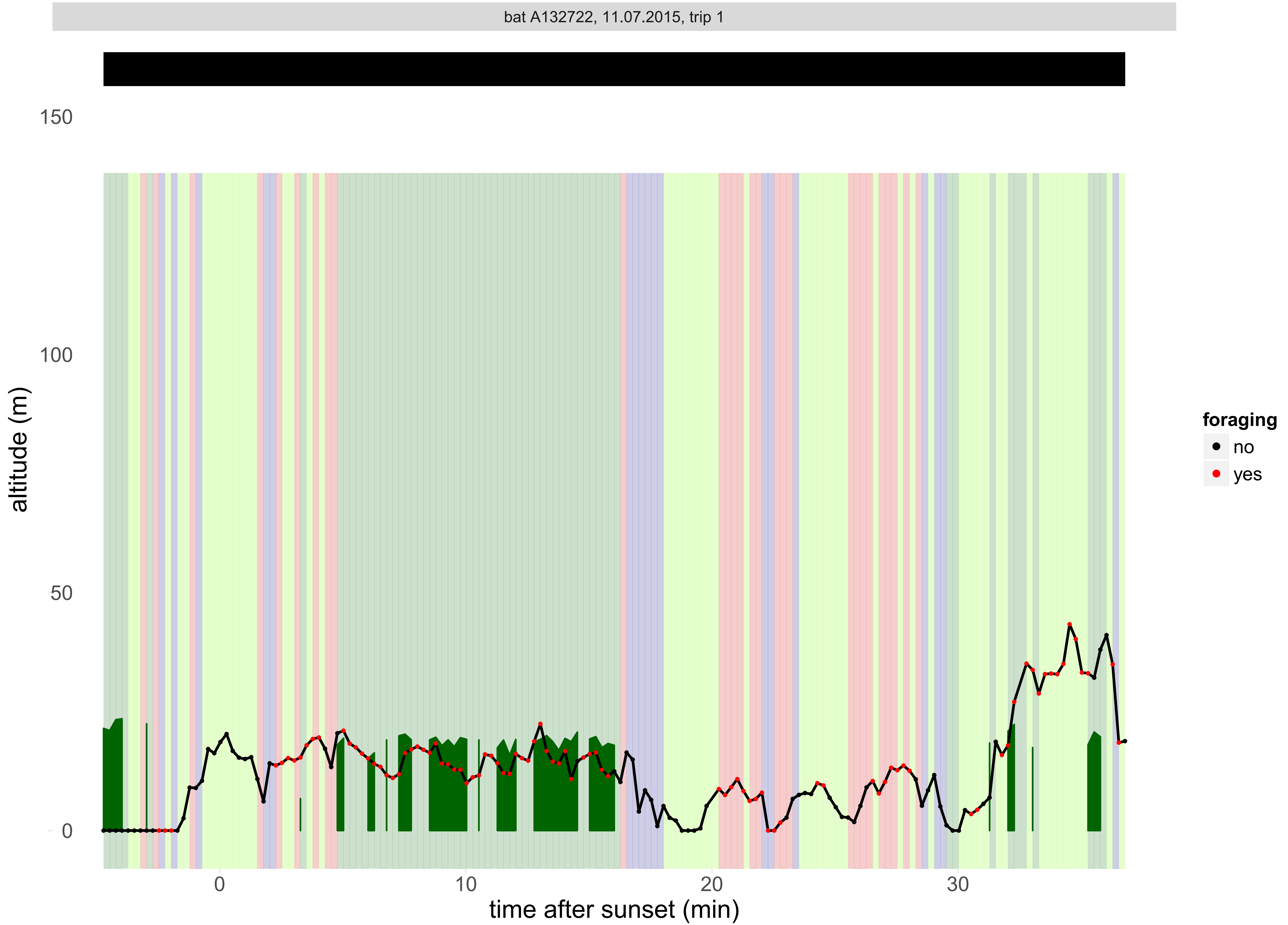

bat A132722, 11.07.2015, trip 1
altitude (m)
150
100
50
0
0
10
20
30
time after sunset (min)
foraging
no
yes

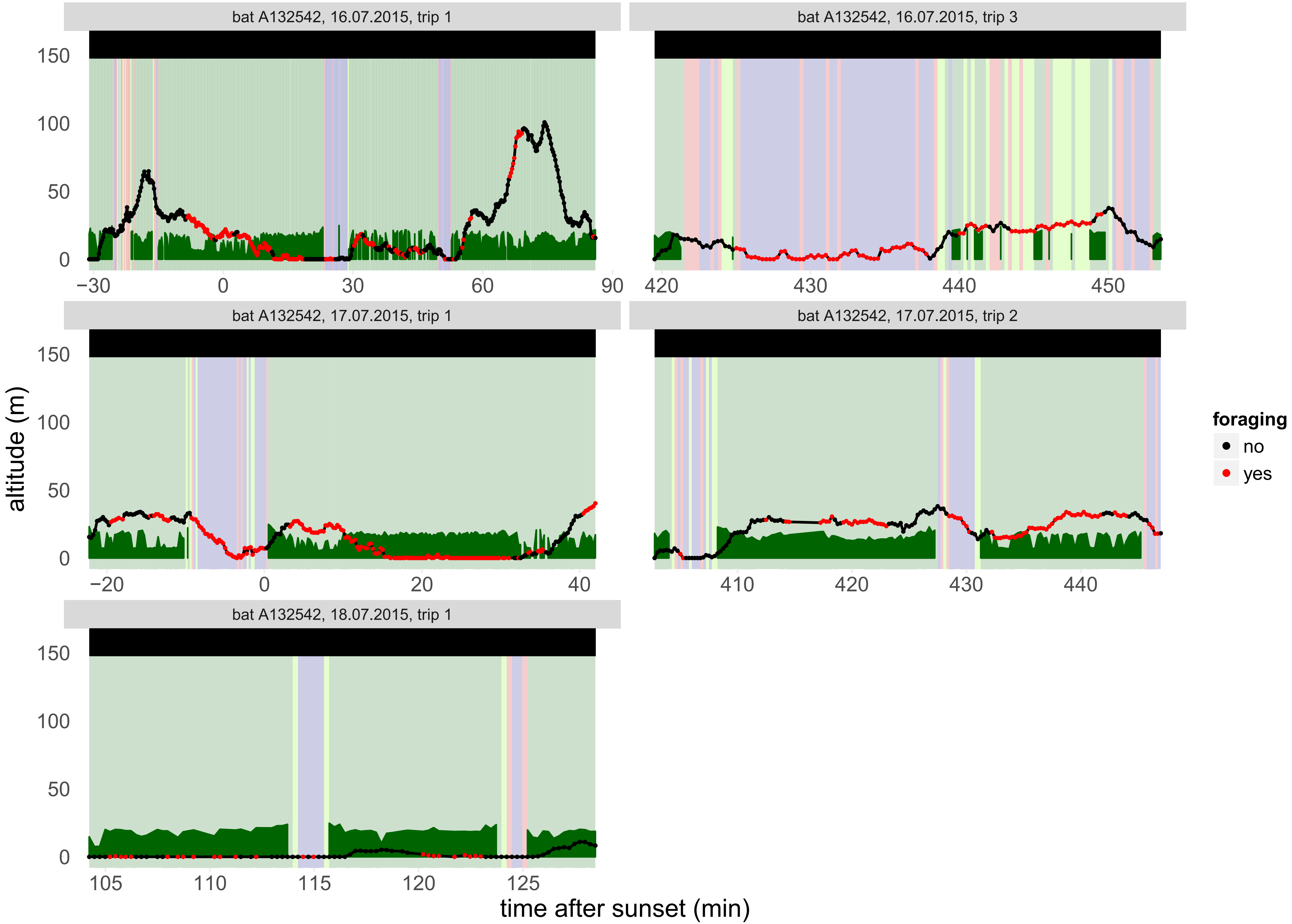
bat A132542, 16.07.2015, trip 1
bat A132542, 16.07.2015, trip 3
bat A132542, 17.07.2015, trip 1
bat A132542, 17.07.2015, trip 2
bat A132542, 18.07.2015, trip 1
altitude (m)
time after sunset (min)
foraging
no
yes
0
50
100
150
−30
0
30
60
90
420
430
440
450
−20
0
20
40
410
420
430
440
105
110
115
120
125

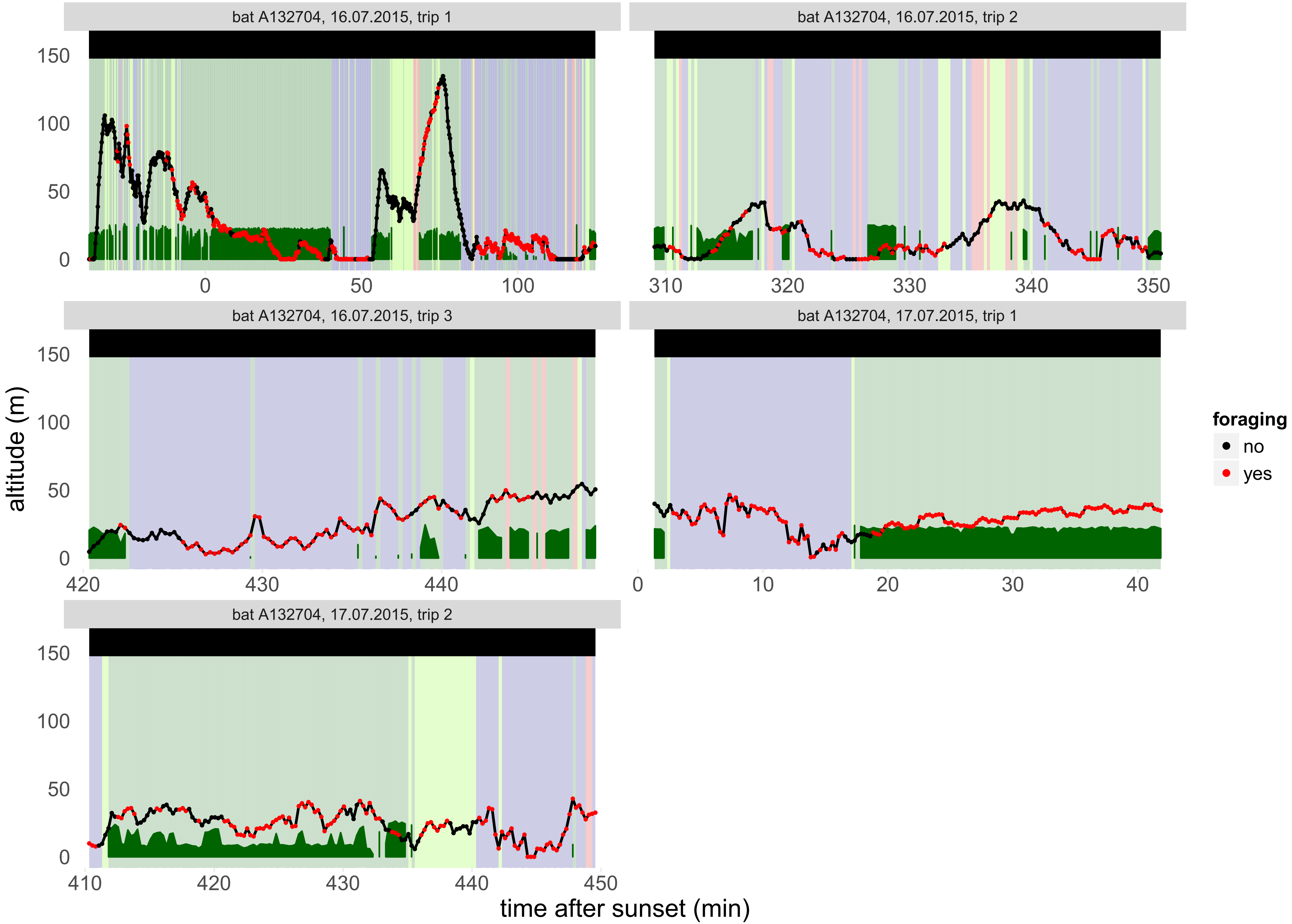

bat A132704, 16.07.2015, trip 1
bat A132704, 16.07.2015, trip 2
bat A132704, 16.07.2015, trip 3
bat A132704, 17.07.2015, trip 1
bat A132704, 17.07.2015, trip 2
altitude (m)
time after sunset (min)
foraging
no
yes

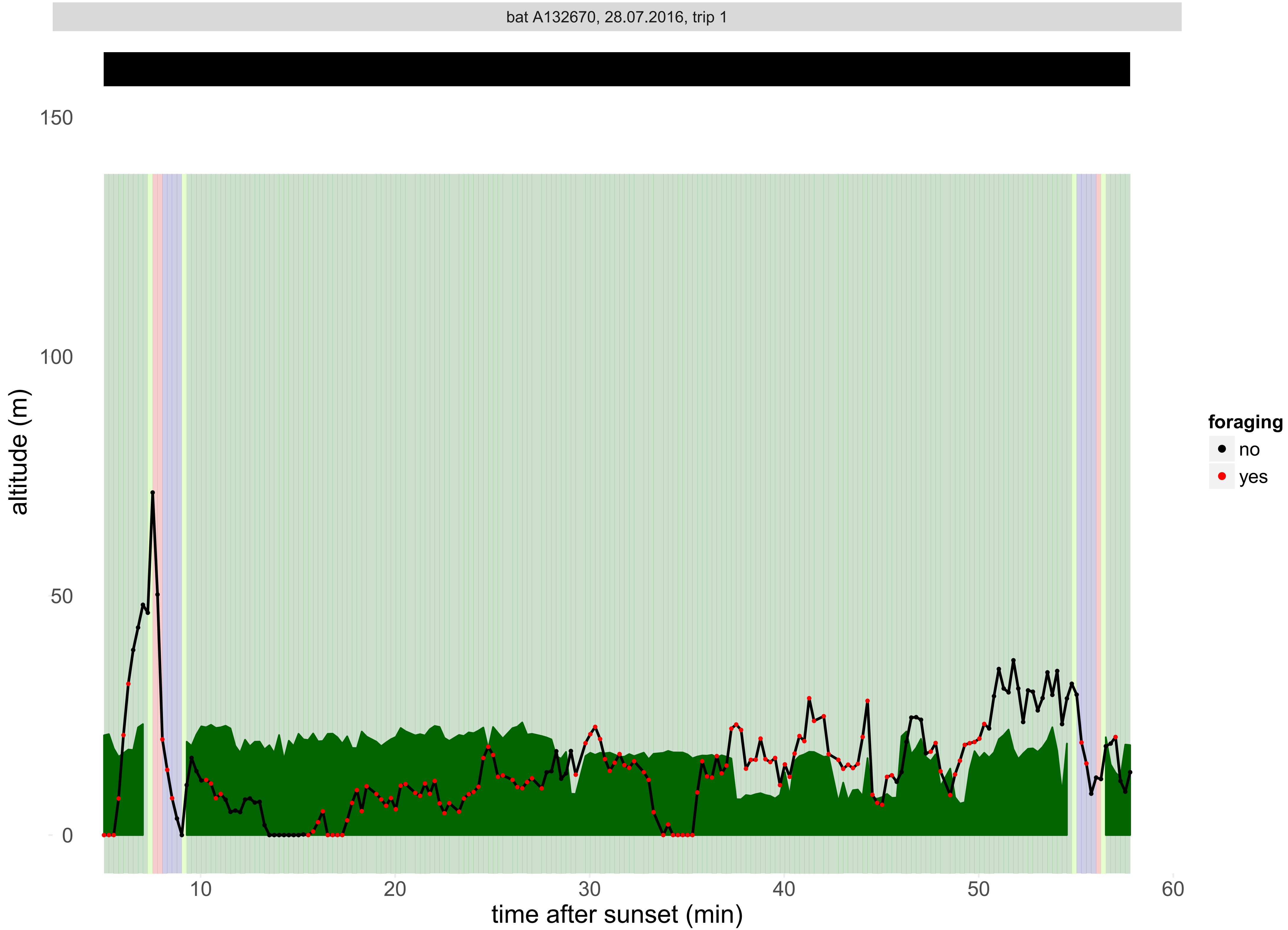

bat A132670, 28.07.2016, trip 1
altitude (m)
150
100
50
0
10
20
30
40
50
60
time after sunset (min)
foraging
no
yes
